# Correction of the X-ray wavefront from compound refractive lenses using 3D printed refractive structures

**DOI:** 10.1107/S1600577520011765

**Published:** 2020-10-19

**Authors:** Vishal Dhamgaye, David Laundy, Sara Baldock, Thomas Moxham, Kawal Sawhney

**Affiliations:** a Diamond Light Source, Harwell Science and Innovation Campus, Didcot, Oxon OX11 0DE, United Kingdom; b Synchrotron Utilisation Section, Raja Ramanna Centre for Advanced Technology, Indore 452012, India; c Department of Chemistry, Lancaster University, Lancaster LA1 4YB, United Kingdom; d Department of Engineering Science, Oxford University, Parks Road, Oxford OX1 3PJ, United Kingdom

**Keywords:** X-ray optics, wavefront correction, X-ray lenses, 3D printing, knife-edge imaging

## Abstract

A refractive phase corrector optics is proposed for the compensation of fabrication error of X-ray optical elements. Here, at-wavelength wavefront measurements of the focused X-ray beam by knife-edge imaging technique, the design of a three-dimensional corrector plate, its fabrication by 3D printing, and use of a corrector to compensate for X-ray lens figure errors are presented. A rotationally invariant corrector was manufactured in the polymer IP-S^TM^ using additive manufacturing based on the two-photon polymerization technique. The fabricated corrector was characterized at the B16 Test beamline, Diamond Light Source, UK, showing a reduction in r.m.s. wavefront error of a Be compound refractive Lens (CRL) by a factor of six. The r.m.s. wavefront error is a figure of merit for the wavefront quality but, for X-ray lenses, with significant X-ray absorption, a form of the r.m.s. error with weighting proportional to the transmitted X-ray intensity has been proposed. The knife-edge imaging wavefront-sensing technique was adapted to measure rotationally variant wavefront errors from two different sets of Be CRL consisting of 98 and 24 lenses. The optical aberrations were then quantified using a Zernike polynomial expansion of the 2D wavefront error. The compensation by a rotationally invariant corrector plate was partial as the Be CRL wavefront error distribution was found to vary with polar angle indicating the presence of non-spherical aberration terms. A wavefront correction plate with rotationally anisotropic thickness is proposed to compensate for anisotropy in order to achieve good focusing by CRLs at beamlines operating at diffraction-limited storage rings.

## Abstract 2

The knife-edge imaging-based wavefront-sensing technique is used for wavefront error characterization of Be X-ray lenses with and without corrector plate. Different 3D printed corrector plates are proposed to achieve pseudo-perfect compensation of wavefront errors of Be X-ray lenses.

## 1. Introduction   

Phase error correction in X-ray optics is a fast-evolving area of enabling technology to generate pseudo perfect optics. The correction introduced by a suitable scheme converts an aberrated optics to pseudo-perfect optics which otherwise prevents achieving diffraction-limited focusing. A few schemes such as active bimorph mirrors (Mimura *et al.*, 2010 ▸), refractive correctors (Sawhney *et al.*, 2016 ▸; Seiboth *et al.*, 2017 ▸), invariable-multilayer deposition (Matsuyama *et al.*, 2018 ▸), diffractive wavefront correction (Probst *et al.*, 2020 ▸) and layer stress controlling method (Cheng & Zhang (2019 ▸) have been demonstrated as tools for phase error corrections of different X-ray optical elements. Refraction-based correctors are thin, easy to insert into the beam path, do not change the optical axis and are straightforward to align. X-ray LIGA fabricated SU-8 wavefront correctors were used in the wavefront error compensation of X-ray mirrors (Laundy *et al.*, 2017 ▸) and X-ray LIGA fabricated lenses (Sawhney *et al.*, 2019 ▸) in one-dimensional (1D) focusing geometry. A silica refractive phase plate manufactured by the laser ablation process was instrumental in reducing the phase error of two-dimensional (2D) focusing Be CRLs (Seiboth *et al.*, 2017 ▸). In each case, the wavefront error is reduced due to the use of suitable correctors, and our group recently demonstrated r.m.s. wavefront error compensation down to the order of λ/100 (Laundy *et al.*, 2019 ▸).

Nano- and micro-fabrications have played a pivotal role in the development of novel micro X-ray optical elements which led to a significant advance in achieving nano- and micrometre-size focused X-ray beams (Li *et al.*, 2020 ▸; Dhamgaye *et al.*, 2014 ▸; Lyubomirskiy, Boye *et al.*, 2019 ▸; Yan *et al.*, 2014 ▸). Highly sensitive X-ray optical measurements, especially sensitive wavefront error measurements, were possible due to microfabrication of 1D and 2D X-ray gratings (Liu *et al.*, 2018 ▸; Weitkamp *et al.*, 2005 ▸; Rutishauser *et al.*, 2012 ▸). Lithography techniques including Si etching, X-ray lithography and laser ablation were used in the development of nano-focusing lenses and wavefront corrector plates. Additive manufacturing or three-dimensional (3D) printing technology is developing rapidly and is revolutionizing many key areas of industries and research. The 3D printer is based on the two-photon polymerization process (Photonic Professional GT2 datasheet, https://www.nanoscribe.com/fileadmin/Nanoscribe/Solutions/Photonic_Professional_GT2/DataSheet_PPGT2.pdf, NanoScribe GmbH), which is capable of patterning arbitrary 3D shapes with micrometre or nanometre resolution. This printer was employed in many state-of-the-art device developments (Dietrich *et al.*, 2018 ▸) and was recently used in X-ray optics developments (Sanli *et al.*, 2018 ▸; Petrov *et al.*, 2017 ▸; Lyobomirskiy, Koch *et al.*, 2017 ▸). The same 3D printer is used for the development of corrector plates.

X-ray optical elements, X-ray mirrors based on reflection, X-ray lenses based on refraction and X-ray zone-plate/multilayer Laue lenses based on diffraction principles are used for micro- or nano-focusing of the X-rays (Ice *et al.*, 2011 ▸). The refractive index (*n*) in the X-ray region for X-rays with energy *E* is
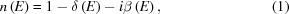
where 1 − δ is a real term, δ is the index of refractive decrement (10^−5^ to 10^−7^) and β is an imaginary term that causes absorption (10^−7^ to 10^−9^). The real part of *n* is slightly less than unity in the X-ray region for all materials, thus the shape of the X-ray lenses is concave, in contrast to convex used in the visible region. Due to weak refraction power, multiple X-ray lenses are used in series by compounding the refraction power of the lenses to achieve a reasonable focal length. Such X-ray lens assemblies are known as compound refractive lenses (CRLs) (Snigirev *et al.*, 1996 ▸). Parabolic-shaped 2D focusing X-ray lenses are fabricated in Be or Al by the mechanical punching method, and 1D focusing lenses in Si, diamond or polymer are manufactured by lithography techniques. Lens fabrication errors result in a deviation of the X-ray pathlength in the lens from the ideal parabolic function. This causes a perturbation of the X-ray wavefront which, when propagated to the focal plane, degrades the focus. With respect to Be CRL fabrication, factors such as mechanical punching (two punching tools with angular or spatial error), density variation, or variation in the chemical composition of the material are responsible for the origin of wavefront errors. The intensity distribution or wavefront errors of given optics are measured by a suitable wavefront-sensing technique. Zernike polynomial fitting is a useful tool in diagnosing visible optics wave aberrations over a circular or annular aperture. Zernike polynomials expansion is used over the wavefront error map in quantifying optics aberrations present in X-ray optics (Celestre *et al.*, 2020 ▸; Seiboth *et al.*, 2016 ▸; Zhou *et al.*, 2018 ▸). An imperfect optics produces blurred images of a source, and the performance improvement of optics by aberration compensation schemes can be expressed in terms of reduction in the coefficient of classical primary (Seidel) optics aberrations closely represented by low-order Zernike polynomials.

Recently (Seaberg *et al.*, 2019 ▸), a 3D printed phase plate in IP-S resist was used to correct wavefront errors of 20 Be lenses, and wavefront analysis was carried out using three wavefront reconstruction techniques for X-ray free-electron laser (XFEL) sources. This paper describes the use of the knife-edge imaging-based wavefront-sensing technique to determine wavefront errors from two different stacks of Be lenses. This wavefront sensing technique is described in our previous work (Laundy *et al.*, 2019 ▸). The optical characterization of a rotationally invariant profiled polymer corrector plate manufactured by 3D printing was carried out at the Diamond Test beamline. After correction with the phase plate, the r.m.s. wavefront error of 98 Be lenses showed a reduction by a factor of six. The knife-edge imaging-based wavefront-sensing technique was originally developed to measure 1D wavefront error profiles but in the present studies it was adapted to measure the full rotational variant wavefront error profiles of the Be CRL. The previously reported study (Seiboth *et al.*, 2017 ▸) used a rotationally invariant corrector and showed a reduction of spherical deformation of the Be lenses. The present study reports the existence of a range of lower- and higher-order optics aberrations in Be CRLs including spherical aberration, astigmatism and coma. We highlight, particularly, that it is impossible to correct all optics aberrations of X-ray lenses with a rotationally invariant corrector when rotationally variant wavefront errors are present. A case study of the effect of rotationally invariant corrector plates versus rotationally variant corrector plates on the corrected wavefront error is described and evaluated in terms of Zernike polynomials. The r.m.s. wavefront error is used to characterize the aberration level from the optics. X-ray lenses differ from visible-light lenses in having weak refraction and strong absorption. This limits the numerical aperture to of order ∼10^−3^. We present a modified form of the r.m.s. wavefront error with weighting due to transmitted intensity.

## 2. Optical characterization setup   

Diamond’s Test beamline B16 was used for at-wavelength characterization of the X-ray lenses and 3D printed corrector plate (Sawhney *et al.*, 2010 ▸). A typical experimental setup used for the wavefront error measurement of CRLs is shown in Fig. 1 ▸. The monochromatic beam from a Si(111) double-crystal monochromator was focused by X-ray lenses and observed on an X-ray detector placed at a distance of ∼1–2 m downstream of the lens’s focus. A corrector plate mounted on an alignment stage was positioned in front of the Be CRLs. The Be CRLs (fabricated by RX Optics), knife-edge (fabricated by X-ray LIGA at ANKA synchrotron) and X-ray detectors (Mini-FDS from Photonics Science and PIPS diode) were mounted on stable rigid platforms. A CRL consisting of *N* = 98 individual Be bi-concave parabolic-shaped lenses, 200 µm radius of curvature at the apex and theoretical focal length 673 mm (image distance *q* = 696 mm) at 15 keV was installed. We will refer to this CRL set as CRL1. A 2D pixel area detector Mini-FDS of pixel size 6.45 µm was used to record the images as a function of knife-edge position. X-ray transmission of the corrector plate was measured using a PIPS diode. A second set of X-ray lenses with *N* = 24 (referred to here as CRL2) was characterized in the same setup with revised positions of knife-edge and detectors from the centre of the CRL.

The wavefront error measurement involved recording the X-ray intensity at the pixel detector as a knife-edge is translated across the focal plane intersecting the focus. The polar coordinates geometry used for knife-edge imaging-based wavefront sensing is shown in Fig. 1 ▸(*b*). The 1D wavefront error along the diameter of the lenses, *i.e.* the central line of the shaded area inclined at an angle, was measured by orientating the knife-edge at the same angle and collecting intensity data of the recorded image from a narrow strip of pixels tilted at the same angle. The wavefront error measured along the diameter was resolved into two radial functions (0 to *r*) separated by 180° around the polar axis, *i.e.* two radial wavefront profiles at 45° and 225° for a 45° knife-edge orientation. The centre of the lenses is located on the detector as the position of maximum intensity transmission. A wavefront error that is constant as a function of radial distance of the entire polar angles is called a rotationally invariant wavefront error. Similarly, a wavefront error that varies as a function of entire polar angle as well as radial distance is called a rotationally variant wavefront error. The optimum performances of the 3D printed corrector plate were analysed by comparing the r.m.s. wavefront errors of Be lenses with and without a corrector plate and comparing focused beam sizes in two orthogonal planes near to the optics focal plane. We define the r.m.s. wavefront error for the lenses over its aperture of radius *R*_0_ weighting with the transmitted intensity as
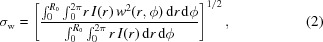
where the wavefront error *w*(*r*,ϕ) is weighted by the X-ray intensity *I*(*r*) which, for uniform incident intensity over the lens aperture *I*_0_, is given by the linear absorption coefficient [μ(*E*)],
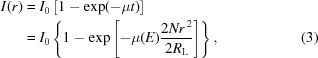
where *R*_L_ is the radius of curvature of the lens, *E* is the X-ray energy, *N* is the number of the lenses, and the wavefront error *w*(*r*,ϕ) is defined on an aperture 0 ≤ *r* ≤ *R*_0_ and 0 ≤ ϕ ≤ 2π.

The wavefront error over a circular aperture can be expressed as a series of Zernike polynomials as functions of the normalized radial position *r*/*R*_0_ and radial angle 0–2π. These are a complete set of basis functions that are orthogonal over a circle of unit radius and are commonly used to represent optical aberrations (Born & Wolf, 1999 ▸). The Zernike polynomials in the Noll notation which uses a single index *j* are defined as (Noll, 1976 ▸)
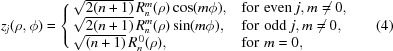
where *m* is the azimuthal frequency, *n* is the radial degree, ρ = *r*/*R*_0_ and
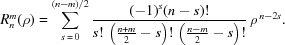
The index *j* is the mode ordering number which is expressed in terms of *n* and *m*. The Zernike polynomial modes (*Z*_*j*_) expansion of the Be lens arbitrary wavefront error is expressed as 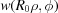 = 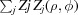 where *Z* *j* is the Zernike coefficient for each *Z*_*j*_ obtained from
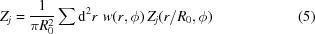
and 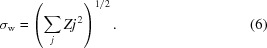
The Zernike polynomial Python library provided by Fan (2019 ▸) is used for fitting lens wavefront errors and determining Zernike coefficients.

## 3. Corrector plate design   

Measurement of the figure error distribution in the Be CRLs is required for the design of a corrector plate. An ideal coherent wavefront from the source at 47 m upstream was considered at the entrance of the Be CRLs. For ideal lenses, an emerging wavefront at the exit of the lenses will be a converging spherical wavefront radius centred on the focus. In reality, the emerging wavefront from the Be CRLs is distorted by variation of lens thickness from the ideal parabolic profile caused by imperfect manufacturing. Other factors such as impurity in the lens material or non-uniform pressed lens material during manufacturing leading to density variations contribute to the origins of wavefront errors of the optic. A knife-edge imaging technique is used for the first time for the investigation of figure error distribution in Be CRLs. This technique reproduces measurements for the particular optics, and wavefront errors recorded for Be lenses are found on a similar order as measured by the other techniques, *e.g.* ptychography or speckle tracking (Seaberg *et al.*, 2019 ▸). The 1D measured wavefront errors along vertical and horizontal lines are shown in Fig. 2 ▸ for four different polar angles as a function of radial position. The lenses are randomly oriented in the casing and show different wavefront error functions at different polar angles. An invariant wavefront profile around the polar axis is evident for the polar angles 90°and 270° (green solid and dashed lines in Fig. 2 ▸) which was measured whilst the knife-edge was stepped along the horizontal line. However, we considered an average error profile calculated from the error profiles measured at different polar angles (solid blue line) and the same error is converted into the design of a rotationally invariant corrector. An optical path length difference (Δ*w*) is introduced by a material of thickness (*t*) with phase error (θ),
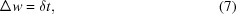
where 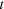 = 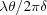 and δ is the refractive decrement of the X-ray refractive index (*n*) given in equation (1). The ratio δ(*E*)/β(*E*) can be used as a selection criterion for choosing a corrector plate material with higher ratio of refraction power to X-ray absorption. Thus, low-atomic-number materials are preferred over higher-atomic-number materials. Materials such as Be, Al, Si, diamond and polymers composed of carbon, hydrogen and oxygen are commonly used for micro X-ray optical elements. A polymer-based corrector plate is used in the present study and its thickness required for compensation wavefront error is calculated using equation (7). A typical 3D printable polymer IP-S of thickness difference Δ*t* = 10 µm will produce a phase advance 2πδΔ*t*/λ and will introduce an optical path difference of 11.74 pm [molecular formula C_14_H_18_O_7_, density = 1.2 g cm^−3^ (Lyubomirskiy, Koch *et al.*, 2019 ▸)]. An estimated thickness profile of the IP-S corrector in a 3D symmetry is shown in Fig. 2 ▸(*b*). Many 3D printers based on fusion deposition modelling or stereolithography produce structures with feature size >>1 µm with a high degree of porosity in the fabricated structure. A nanoscribe 3D printer is an ideal tool for 3D printing of the corrector plate (Nanoscribe GmbH, Germany). It is capable of printing arbitrary features with sub-micrometre precision in three dimensions. The surface finish of the printed structure is ∼20 nm which is good for the normal-incidence optics used in the X-ray region (Photonic Professional GT2 datasheet, https://www.nanoscribe.com/fileadmin/Nanoscribe/Solutions/Photonic_Professional_GT2/DataSheet_PPGT2.pdf).

The design of the corrector plate was prepared in AutoCAD and converted into a 3D CAD-step file. A corrector plate was fabricated using Nanoscribe Photonic Professional GT2 (Nanoscribe GmbH, Germany) at Lancaster University, UK. The dip in lithography mode was used to print the 3D design in IP-S (Nanoscribe GmbH). The laser source used for printing was a femtosecond Ti-sapphire type (800 nm, 80 MHz, 50 fs). IP-S was drop-cast onto ITO (75–100 Ω^2^) coated glass N1.5 thickness coverslips (Diamond Coatings Ltd, Halesowen, UK). The resist was exposed from bottom to top using a femtosecond laser pulse focused in voxel by 25× objective with laser power 55% and writing speed 200000 µm s^−1^. Patterned IP-S resist was developed in PGMEA for 20 min, rinsed in IPA for 5 min and dried using N_2_ enriched air.

## 4. Results and discussions   

### 4.1. Rotationally invariant wavefront errors measurement and its compensation   

The effectiveness of the corrector plate in the wavefront error compensation depends on various factors such as repeatability of wavefront measurements between successive beam times, the stability of the optics/beam, design of the corrector plate, fabrication errors in the corrector plate and alignments of the corrector plate to the CRL optic axis. A rotationally invariant 3D printed corrector was placed upstream to the Be CRL as shown in Fig. 1 ▸ for the figure error corrections of the Be CRL. The wavefront errors of Be CRL1 were measured and the repeatability in the measurements was confirmed by comparing the measurement with that made during the design of the corrector plate. Good alignment of the centre of the corrector plate relative to the lens optical axis in a beam path is critical in achieving optimum compensation results. With the corrector plate position in the nearly plane wavefront before the focusing lenses, the correction is insensitive to the correctors’ longitudinal position. The lateral position of the phase plate is more important, with good alignment to the axis of the lens being required. To achieve this, the phase plate was stepped laterally within the lens aperture with coarser 5 µm and finer 1 µm step size and the corresponding r.m.s. wavefront error was determined using equation (2). The best lateral positions for the corrector plate are achieved by minimizing the r.m.s. wavefront error in the respective planes.

An average of the CRL1 wavefront errors measured at four different polar angles before and after the corrections is shown in Fig. 3 ▸. The r.m.s. wavefront error [equation (2)] is found to be 14.4 pm before the correction and 2.4 pm after the correction which is an improvement by a factor of six. The expected performance of a designed corrector plate is shown as ‘after correction (calculated)’ in Fig. 3 ▸ which is obtained by subtracting the wavefront error values used for the design of the corrector plate (dashed magenta) from the corresponding error values measured for CRL1 before correction (blue). The r.m.s. wavefront error difference between the designed corrector (discussed in Section 2) and the fabricated corrector is <1 pm. This difference is due to various contributions such as infidelity in corrector fabrication, alignment/stability of optics and repeatability in the wavefront measurements. X-ray absorption by the corrector was calculated by measuring the PIPS diode photocurrent for direct beam and placing the corrector plate in the beam path. The transmission of the corrector plate was found to be ∼99%. A clear improvement in the focus profiles in the vertical and horizontal direction was observed after the introduction of the corrector [Figs. 4 ▸(*a*) and 4 ▸(*b*)]. The focus profiles, before and after corrections, are measured at the same focal distance from the centre of the CRL. The corrector plate has improved the vertical (horizontal) focus size to 0.9 µm (2.5 µm) from 2.3 µm (3.7 µm) due to the aberrated wavefront. The focus size of CRLs at a bending-magnet source is limited by the size of the de-magnified source.

A type of wavefront aberration exists in CRL1 before and after the corrections were quantified using Zernike polynomials expansion up to order *n* = 16. Fig. 5 ▸ shows the amplitude of the first 36 Zernike coefficients and coefficients corresponding to higher-order spherical aberrations only (*Z*37, *Z*56, *Z*79, *Z*106 and *Z*137) as the values of the remaining coefficients of the higher orders are either small or zero. Zernike polynomial coefficients *Z*1 to *Z*4 are not aberrations but they describe the surface positioning. *Z*1 is constant over the whole aberration map and therefore not considered. The misalignment of optics is expressed in the system tilts *Z*2 and *Z*3 along two orthogonal planes and term *Z*4 defines defocusing. The major optics aberrations observed in the Be CRLs were due to primary (*Z*11), secondary (*Z*22), tertiary (*Z*37) and higher-order spherical aberrations. These spherical deformations were well corrected after the introduction of the corrector plate. The defocus term (*Z*4) observed was caused by the displacement of the knife-edge from the focal plane in the direction along the optical axis. This study does not show a contribution from non-spherical aberration terms. The r.m.s. wavefront error is given as the sum of squares of all Zernike coefficients. The r.m.s. calculated by considering all Zernike coefficient values except (*Z*1–*Z*4) is 14.2 pm before correction and 2.7 pm after correction. These values match well with the ones calculated using equation (2).

### 4.2. Rotationally variant wavefront errors measurement and its compensation   

We extended our investigation to another set of lenses: CRL2 (*N* = 24). We investigated the polar-angle-resolved wavefront error distributions by making wavefront measurements with the knife-edge rotated in angles about the optical axis to obtain the radial wavefront error over the polar angle from 0° to 360°. The intensity recorded in a 2D pixel detector was processed only for those pixels that lie along a line inclined at a rotated angle. Unfortunately, the knife-edge scan data is not complete for CRL1 as the measurement script failed twice during the experiments. An average radial wavefront error calculated over a complete radial profile was used for missed measurements at the polar angles (135–165° and 315–345°). Figs. 6 ▸(*a*) and 6(*b*) show polar plots of the wavefront errors in both CRLs before correction. The wavefront errors of both CRLs are close to being invariant but show anisotropic wavefront error distributions in the polar angles. The distributions are not radially concentric but approximately oval, rotated at 45° and 90° for CRL1 and CRL2, respectively. An analytical approach was considered to evaluate the performance of the rotationally invariant corrector plate in compensating for the rotationally variant wavefront errors of CRL1 and CRL2. The remaining wavefront errors after correction by the rotationally invariant corrector plates are shown in Figs. 6 ▸(*c*) and 6(*d*).

The uncorrected wavefront errors of both CRLs were found in a similar range. We noticed no per-lens wavefront error accumulation – otherwise peak-to-peak wavefront errors of CRL1 would be four times higher than for CRL2 over the whole lens aperture. This observation is true near the optical axis of the lenses where maximum transmission of the X-rays is observed. Any rotation of the individual lens in the lens casing may be averaging figure errors and such averaging is apparent more in CRL1 compared with CRL2.

The wavefront error surfaces shown in Figs. 6 ▸(*a*)–6(*d*) were fitted with Zernike polynomials, and corresponding amplitudes of Zernike coefficients are shown in the bar chart in Fig. 7 ▸. To avoid areas of non-measurements in the fitting and obtain a good fit, a radial distance (*R*_0_) of ±186 µm for CRL1 and ±305 µm for CRL2 from the centre of the wavefront error map was chosen. The strength of various optics aberration expressed by Zernike polynomials expansion before and after corrections shows the existence of lower and higher orders of spherical and non-spherical optics aberrations. As discussed in the previous section, here too spherical aberrations of both CRLs are compensated well by the rotationally invariant corrector plate. However, non-spherical aberration terms (including astigmatism, coma, *etc*.) and higher-frequency terms (trefoil, tetrafoil, pentafoil, hexafoil, *etc*.) remained uncorrected. Astigmatism in CRL2 contributes significantly to the remaining optics aberration which cannot be ignored for obtaining diffraction-limited focusing. The primary optics aberration tilt, defocus, astigmatism, coma, and spherical aberration are expressed in terms of Zernike coefficients (*Z*2, *Z*3), *Z*4, (*Z*5, *Z*6), (*Z*7, *Z*8) and *Z*11, respectively.

We propose two possibilities (case 1 and case 2) for correction of X-ray optics aberrations in CRLs using customized corrector plates. In the first case, a corrector plate is fabricated with a thickness profile in two dimensions that fully corrects the wavefront over the full aperture of the lens. In the second case the spherical terms are corrected using a radially invariant corrector and an additional in-line corrector plate used to correct selected radially variant higher-order terms in the Zernike expansion, such as astigmatic terms.

#### 4.2.1. Case 1   

This has the advantage that complete correction can be achieved with a single corrective element; however, alignment becomes more difficult, as in addition to transverse alignment the corrective optic must also be aligned in rotation angle about the optical axis. It is also necessary to measure the full 2D wavefront error in order to design the profile of the corrector.

We have worked out the designs of such rotationally variant corrector plates exclusively for CRL1 and CRL2. The designs are shown in Figs. 8 ▸(*a*) and 8(*b*) and they can be fabricated by 3D printing. Such corrector plates are planned to be manufactured in the near future and characterized using CRL1 and CRL2 at the Diamond Test beamline. The proposed rotationally variant corrector plate can be extended for the wavefront corrections of CRLs made from Al or polymer materials. An exclusive 3D corrector plate is feasible to build on the same chip in line with the nano-focusing lenses fabricated by either LIGA or semiconductor manufacturing techniques. The 3D correctors made in IP-S polymer are useful at a bending-magnet source, but this polymer degrades quickly in the higher-intensity beams of undulator or XFEL sources. However, searching for a robust material for the corrector plate is necessary to deploy rotationally invariant/variant corrector plates with X-ray optics at beamlines operational on diffraction-limited storage rings or XFELs.

#### 4.2.2. Case 2   

In CRL2, the low-order astigmatism (Zernike polynomials *Z*_5_ and *Z*_6_) contributes almost 50% of the total remaining aberrations after the correction introduced by the radially invariant corrector plate. A customized second corrector plate dedicated to the compensation of low-order astigmatism can be designed in the following way. The wavefront error due to astigmatism is
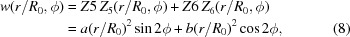
and in the Cartesian form
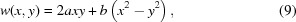
where *Z*5 and *Z*6 are Zernike coefficients for Zernike mode *Z*_5_ and *Z*_6_ and their values are extracted from fitted data (Fig. 7 ▸), and *a* and *b* are constants.

The above wavefront definition creates a parabolic surface in the *xy* plane and it is possible to manufacture using a 3D printer. Fabrication of a sequence of correctors would allow a degree of adaptability to be incorporated into the correction. Table 1 ▸ summarizes the coefficients of the lower-order Zernike polynomials that closely represent classical aberrations, for Be CRLs wavefront errors before and after corrections [‘Corrector1’, rotationally invariant corrector plate; ‘Corrector2’, rotationally variant corrector plate, as defined in equation (9)]. The r.m.s. wavefront error of the optics is reduced from 24.0 pm to 13.3 pm with the first-order correction plate and finally to 4.69 pm (∼0.06λ) with the second-order correction plate. For primary aberrations of CRL2 excluding the piston, tilt and defocus terms, the obtained r.m.s. value is ∼1 pm.

## 5. Conclusions   

The knife-edge imaging wavefront-sensing technique was successfully used in X-ray lenses wavefront error measurements and the optical characterization of a 3D printed corrector. The use of a rotationally invariant 3D printed wavefront corrector plate in wavefront errors compensation of 98 Be X-ray lenses was demonstrated. The r.m.s. wavefront error of rotationally invariant wavefront aberrations in Be CRLs was reduced by 84% after the introduction of a rotationally invariant 3D printed corrector. Zernike polynomials analytical fitting is useful in the quantification of optics aberrations before and after correction wavefront errors. All orders of spherical aberrations are found corrected after the insertion of a rotationally invariant corrector plate but it is apparent that significant non-spherical aberrations still remain. Thus, a rotationally invariant corrector plate is unable to completely compensate optics aberrations CRLs. The knife-edge imaging technique was adapted to measure the full 2D wavefront errors of two X-ray lenses sets CRL1 and CRL2. The Zernike polynomial fitting of measured wavefront error maps of CRL1 and CRL2 showed the existence of lower- and/or higher-order rotationally invariant and variant optics aberrations. We have therefore specified wavefront corrector plates which could approach complete compensation of the wavefront errors. The role of the present 3D printer technology is important in achieving the precision manufacturing of rotationally variant corrector plates. This is a possible way to tackle optics aberrations in X-ray optics and achieving r.m.s. wavefront error compensation below 0.07λ. The present framework of wavefront measurement and corrections is useful in X-ray optics being used at the third- and fourth-generation synchrotron facilities and XFELs.

## Figures and Tables

**Figure 1:**
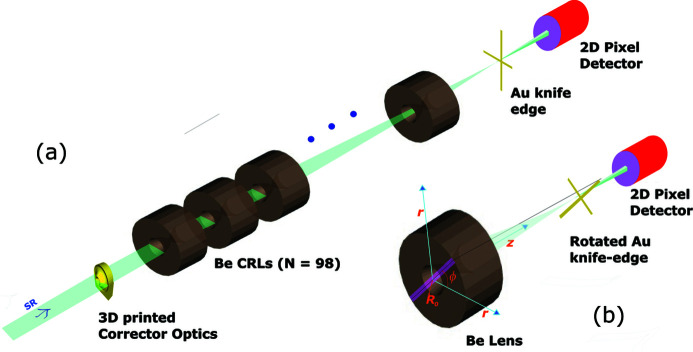
(*a*) Schematic experimental setup at B16 Test beamline, Diamond Light Source. (*b*) The geometry used for rotationally variant wavefront error measurements; a knife-edge was rotated at an angle (*e.g.* ϕ = 45°) for the measurement of the corresponding wavefront errors in the lens area highlighted by the pink stripe.

**Figure 2:**
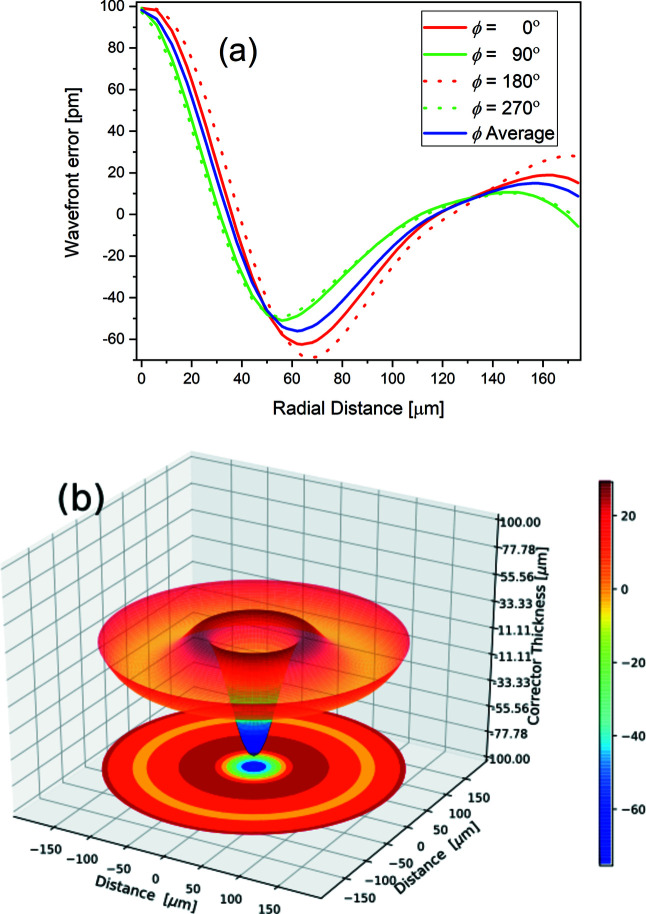
(*a*) Wavefront error distribution of 98 Be lenses in its 2D circular aperture at various polar angles. The solid blue line shows the average wavefront error over polar angles measured and was used for the design of a rotationally invariant corrector plate. (*b*) Schematic 3D design of the corrector plate.

**Figure 3:**
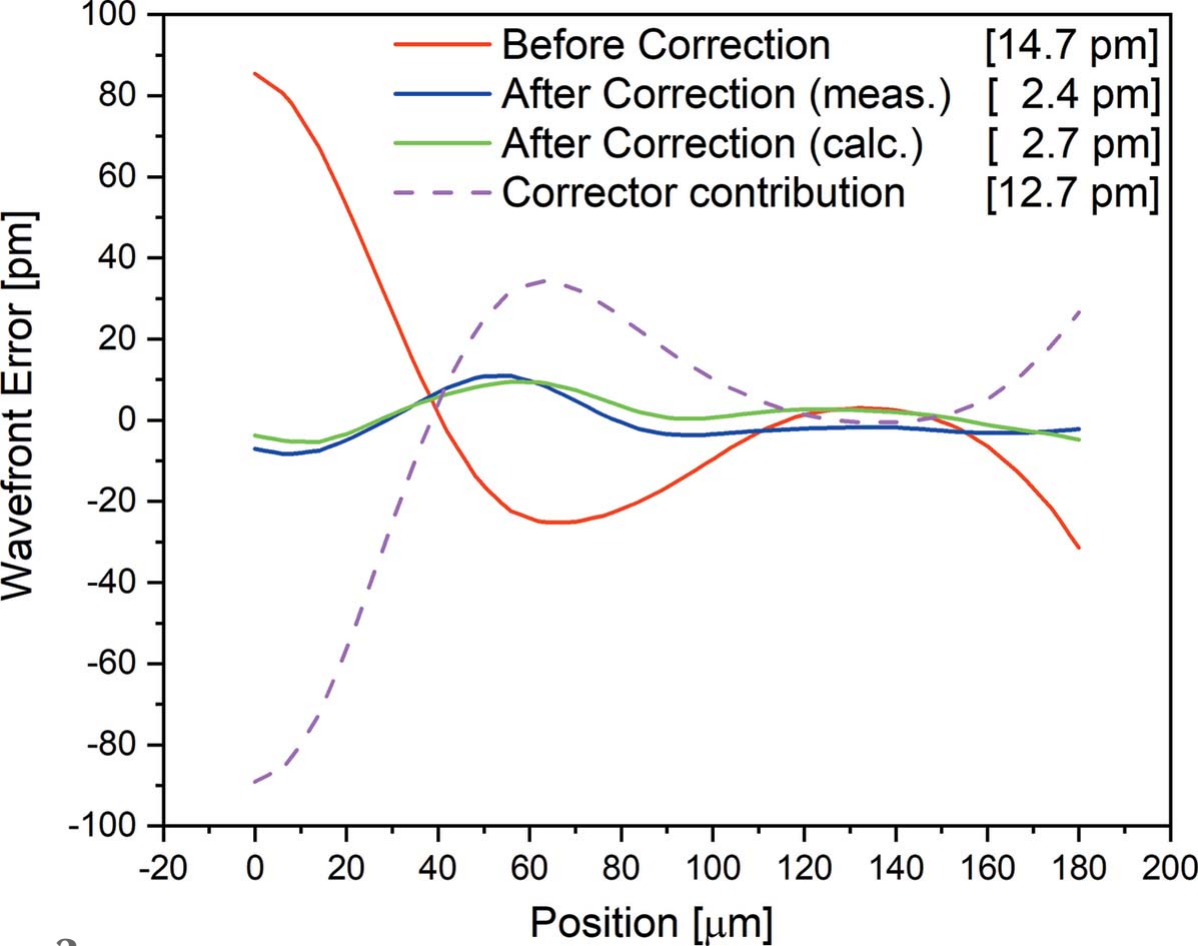
Rotationally invariant wavefront errors of the Be CRLs, before correction, after correction and wavefront error profile by rotationally invariant corrector plate. The r.m.s. wavefront error calculated in each case is given in square parentheses.

**Figure 4:**
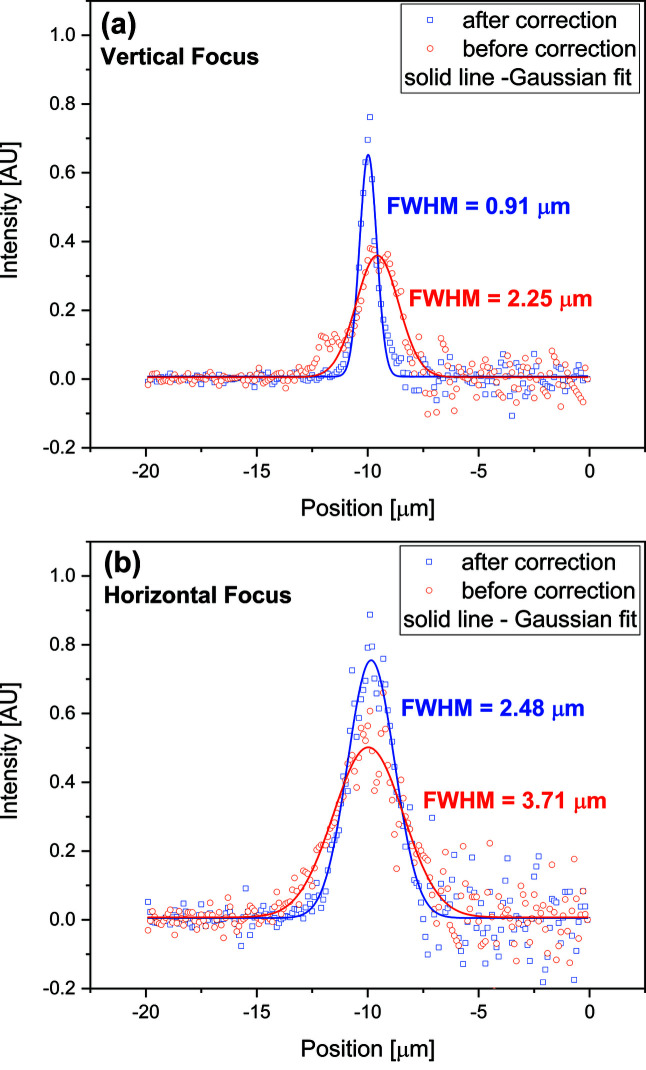
Improvement in imaging the B16 bending-magnet source in the (*a*) vertical and (*b*) horizontal direction after insertion of the corrector plate. Solid lines show a Gaussian fit for the corresponding focus profile measurements.

**Figure 5:**
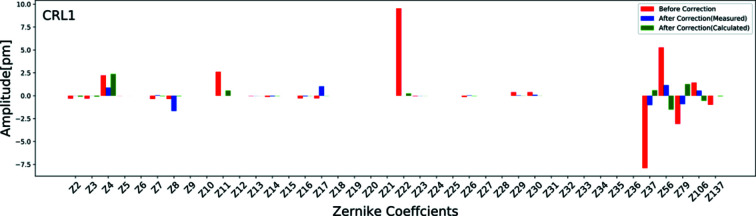
Zernike polynomial fitting over measured and averaged wavefront errors of CRL1 before and after correction (measured and calculated corrector plate contribution).

**Figure 6:**
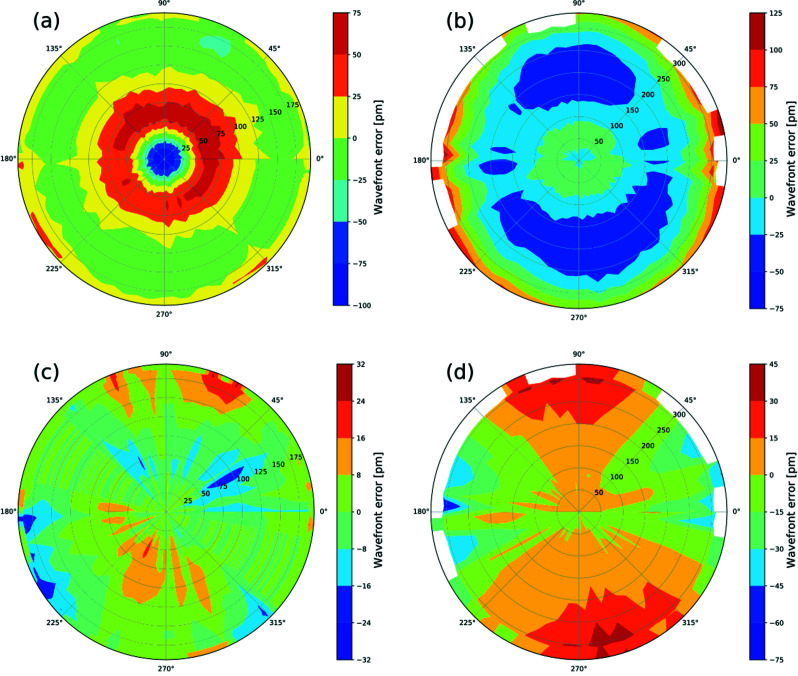
Polar plots showing rotationally variant wavefront errors of Be CRLs (*a*, *b*) and after correction using rotationally invariant corrector plates (*c*, *d*) for CRL1 and CRL2, respectively.

**Figure 7:**
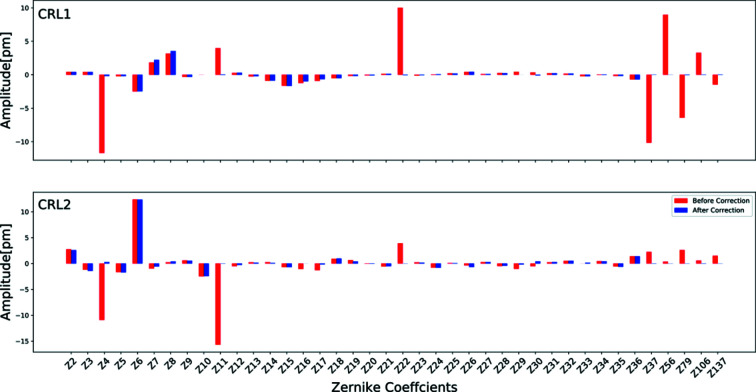
Zernike polynomial fitting over measured rotationally variant wavefront errors of CRL1 and CRL2 before and after correction (calculated rotationally invariant corrector plate contribution).

**Figure 8:**
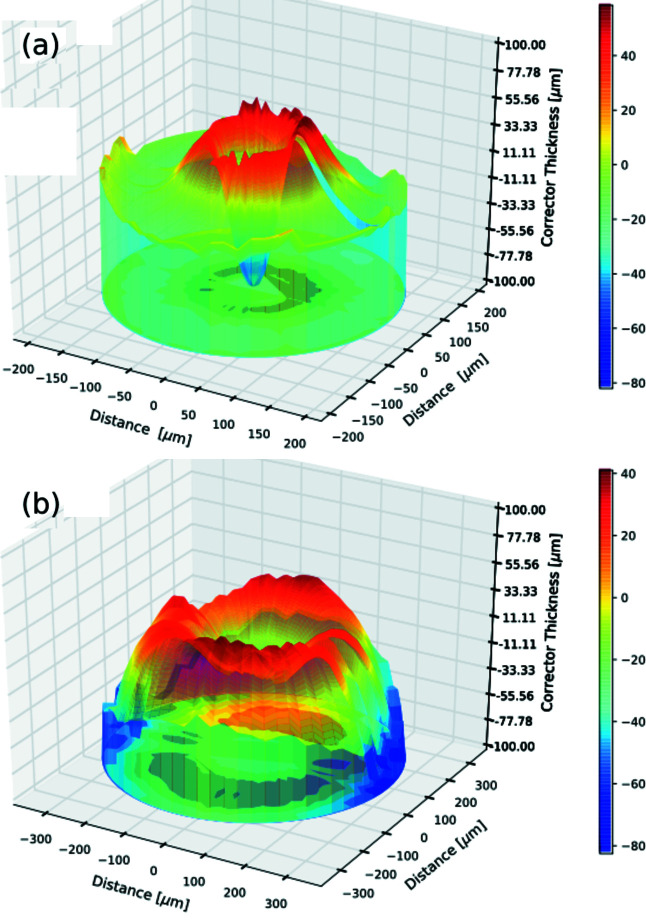
Estimated design of a rotationally variant corrector plate for (*a*) CRL1 and (*b*) CRL2.

**Table 1:** Primary optics aberration in terms of Zernike coefficients for a Be CRL before and after corrections introduced by corrector1 (rotationally invariant corrector) and corrector2 (rotationally variant corrector)

Optics aberrations	Zernike coefficients in Noll notations		
	Before correction (pm)	After correction (pm)	
		Corrector1	Corrector2
Tilt	*Z*2 = 2.8	2.6	2.6
	*Z*3 = −1.2	−1.4	−1.4
Defocus	*Z*4 = −10.9	0.3	0.3
Astigmatism	*Z*5 = −1.7	−1.7	−0.02
	*Z*6 = 12.4	12.4	0.2
Coma	*Z*7 = −1.0	−0.6	−0.6
	*Z*8 = 0.2	0.4	0.4
Spherical aberration	*Z*11 = −15.7	−0.03	−0.03
R.m.s. wavefront error (pm) (lower-order polynomials)	20.1	12.5	0.8
R.m.s. wavefront error (pm) (all order polynomials)	24.0	13.3	4.7
